# Femtosecond quantification of void evolution during rapid material failure

**DOI:** 10.1126/sciadv.abb4434

**Published:** 2020-12-16

**Authors:** James Coakley, Andrew Higginbotham, David McGonegle, Jan Ilavsky, Thomas D. Swinburne, Justin S. Wark, Khandaker M. Rahman, Vassili A. Vorontsov, David Dye, Thomas J. Lane, Sébastien Boutet, Jason Koglin, Joseph Robinson, Despina Milathianaki

**Affiliations:** 1 Department of Mechanical and Aerospace Engineering, University of Miami, Coral Gables, FL 33146, USA.; 2 York Plasma Institute, Department of Physics, University of York, Heslington, York YO10 5DD, UK.; 3 Department of Physics, Clarendon Laboratory, University of Oxford, Parks Road, Oxford OX1 3PU, UK.; 4 Advanced Photon Source, Argonne National Laboratory, Lemont, IL 60439, USA.; 5 Aix-Marseille Université, CNRS, CINaM UMR 7325, Campus de Luminy, 13288 Marseille, France.; 6 Department of Materials, Imperial College, South Kensington, London SW7 2AZ, UK.; 7 DMEM, University of Strathclyde, Glasgow G1 1XQ, UK.; 8 SLAC National Accelerator Laboratory, Menlo Park, CA 94025, USA.

## Abstract

Understanding high-velocity impact, and the subsequent high strain rate material deformation and potential catastrophic failure, is of critical importance across a range of scientific and engineering disciplines that include astrophysics, materials science, and aerospace engineering. The deformation and failure mechanisms are not thoroughly understood, given the challenges of experimentally quantifying material evolution at extremely short time scales. Here, copper foils are rapidly strained via picosecond laser ablation and probed in situ with femtosecond x-ray free electron (XFEL) pulses. Small-angle x-ray scattering (SAXS) monitors the void distribution evolution, while wide-angle scattering (WAXS) simultaneously determines the strain evolution. The ability to quantifiably characterize the nanoscale during high strain rate failure with ultrafast SAXS, complementing WAXS, represents a broadening in the range of science that can be performed with XFEL. It is shown that ultimate failure occurs via void nucleation, growth, and coalescence, and the data agree well with molecular dynamics simulations.

## Teaser

Ultrabright x-rays capture the sequence of material damage accumulation during high strain rate catastrophic failure.

## INTRODUCTION

Material failure mechanisms are rate dependent (*1*), and dynamic material failure occurring at the speed of sound is of broad interest, e.g., from planetary to jet engine debris impacts to laser-driven shocks and their applications in femtosecond machining and laser shock peening. Extremely high tensile strain rates can occur when a decaying shock wave reaches a free surface or an interface of lower shock impedance, generating a counter-propagating rarefaction wave that interacts with the decaying portion of the forward-traveling shock to produce a region of tension in the material. Depending on the magnitude and duration of this tensile stress, the material may fail, and this dynamic phenomenon is termed spallation.

The void formation that leads to high strain rate failure was first examined by incipient spallation experiments performed by Seaman *et al.* (*2*). In these experiments, the applied stresses were below the spall strength of the material, and an incipient spallation zone formed, as opposed to total material failure. The samples were then examined with microscopy, and the microscale voids and cracks were quantified. While these experiments were seminal to our understanding of spallation, the degree of precision from a two-dimensional image may be inaccurate (*3*) and, by definition, does not capture the void distribution at the point of failure itself. Furthermore, the deformation mechanisms at these lower stresses and strain rates may not be representative of the deformation mechanisms under the more extreme conditions and strain rates. Accurate quantification of damage evolution is essential to understanding material failure and is necessary to develop and validate constitutive material failure models.

The recent concurrent advancements in both bright x-ray sources and powerful short-wavelength lasers have made it possible to probe the crystal lattice response with in situ wide-angle x-ray scattering (WAXS) during high strain rate deformation. This technique has been used to examine initial compression after an applied shock, at time scales and length scales that have previously been limited to modeling predictions (*4*–*11*), and has also been used to show that very high transient tensile strains can be sustained by materials at high strain rates (*12*, *13*).

**Fig. 1:**
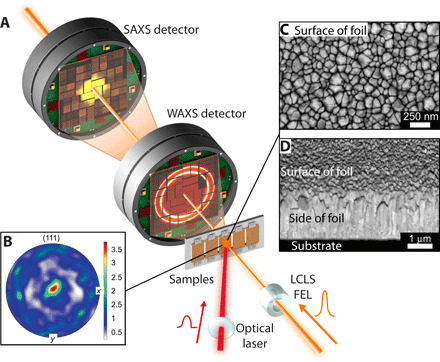
Experimental arrangement at the LCLS and details of the initial Cu foil microstructure. (**A**) Experimental arrangement at LCLS. The XFEL beam impinges the Cu with substrate sample shortly after a laser shock pulse with a “clipped” leading edge. The WAXS data are recorded in the front detector and small angle (SAXS) data recorded in the rear detector. (**B**) (111) pole figure from EBSD; the intensity in the center of the pole figure refers to the foil normal (growth) direction. (**C**) Secondary electron scanning electron microscopy (SEM) imaged normal to the sputtered Cu surface, illustrating the nanoscale planar pores between grains, and (**D**) backscattered electron SEM imaged at a 70° tilt angle, highlighting the columnar grain microstructure through the foil thickness. Graphic design credit: Gregory Stewart, SLAC National Accelerator Laboratory.

In the current work, polycrystalline copper foils are subjected to rapid straining at ∼0.5 × 10^9^ s^−1^ via picosecond laser ablation and probed in situ with 30-fs ultrafast x-ray free electron (XFEL) pulses emitted by the Linac Coherent Light Source (LCLS) (*14*). WAXS and small-angle x-ray scattering (SAXS) data were simultaneously recorded using two Cornell-SLAC Pixel Array Detectors (CSPAD), each with 2.3 megapixels, as shown schematically in Fig. 1A (*15*). SAXS provides scattering patterns from nano- to micrometer differences in composition or density. With this length scale and a two-phase approximation, the technique is of broad importance to the study of material defects (*16*), degradation (*17*), precipitation (*18*), biological studies (*19*), and porosity analysis (*20*–*23*). Thus, the current setup allows high strain rate void evolution to be monitored in the rear SAXS detector and correlated to the lattice strain response (i.e., Bragg diffraction) monitored in the front WAXS detector, and previous experiments have indicated that such measurements should be feasible (*24*–*26*).

The experiment represents the first in situ study of spallation and failure at the ultrahigh strain rates that occur following the initial lattice compression. The spall strength is determined to be approximately 8.5 GPa, measured at a strain rate that is an order of magnitude higher than has been previously reported (*27*–*29*). The SAXS model fits to the in situ experimental data allow quantifiable determination of the failure mechanism, confirming that spallation occurs by the nucleation, growth, and coalescence of voids or cracks (*30*–*32*). This view is supported in the literature by multimillion-atom molecular dynamics (MD) simulations (*33*–*35*).

Multimillion-atom MD simulations were performed in the current work to further develop interpretation and to provide additional confidence in the experimental data. The wide range of spatial and temporal scales of relevance to this work makes a single comprehensive simulation impossible. However, a representative simulation with simplifying assumptions provides good agreement between simulation and microstructure evolution, illustrating a spheroidal void nucleation and growth process.

## RESULTS

### Sample characterization by electron microscopy

The experimental details are presented in Materials and Methods. One-micrometer-thick polycrystalline Cu films were vacuum-deposited on <100>-oriented 500-nm-thick amorphous Si_3_N_4_ substrates. High strain rate material properties are dependent on microstructure (*36*); thus, the samples were characterized in detail with electron microscopy. Sample thicknesses were confirmed, and grains were columnar (Fig. 1D). The mean grain width at the sample surface was ∼80 ± 20 nm with SD (Fig. 1C). Electron backscatter diffraction (EBSD) confirmed a (111) fiber grain texture in the sample growth direction (Fig. 1B).

### Validation of the LCLS SAXS experimental arrangement

The response of the LCLS SAXS detector setup was evaluated with a known standard glassy carbon sample and further cross-calibrated by comparing measurements of the Cu-with-substrate foil that were recorded at the LCLS with measurements that were recorded at the 9-ID SAXS beamline at the Advanced Photon Source (APS).

Glassy carbon standard samples were mounted in the LCLS sample chamber, and scattering measurements were recorded. The azimuthally averaged SAXS data from the glassy carbon sample determined at LCLS were rescaled to match the standard reference measurement, as part of the absolute scattering intensity reduction process. The sample measured at LCLS shows good agreement to the reference measurement between 0.01 and 0.1 Å^−1^, as shown in fig. S1A.

Sputtered Cu with substrate samples were mounted in the beamlines at LCLS and at the 9-ID SAXS/USAXS (ultra-SAXS) instrument at the APS, and scattering measurements were recorded. The processed SAXS data from these samples in the initial condition are in excellent agreement between the LCLS and calibrated APS instruments, shown in fig. S1B. In this figure, for the purpose of data comparison between instruments, the LCLS data for Cu have been multiplied by the ratio in scattering length density contrast at 21 keV (used at the APS) and 8.8 keV (used at LCLS) to account for the difference in x-ray photon energies used between instruments.

### High strain rate scattering data

Sputtered Cu-with-substrate samples were sequentially mounted at the LCLS beamline for in situ simultaneous SAXS/WAXS measurements during high strain rate deformation. The experimental arrangement is shown in Fig. 1A. Shocks were driven through the Cu surface via laser ablation and were simultaneously probed by the XFEL pulses with scattering data recorded in the front (WAXS) and rear (SAXS) detectors. The leading edge of the laser pulse that produced the shock was “clipped” to produce a sharp shock rise time. The pump-probe time delay was increased at picosecond time increments for each sequential measurement, producing a sequence of femtosecond snapshots of the high strain rate deformation process. The front detector recorded WAXS from the {111} reflections, mapping the lattice-level strain response within the material, while evolution of the small-angle scattering spectra was monitored by the rear SAXS detector. Further experimental details are provided in Materials and Methods.

**Fig. 2:**
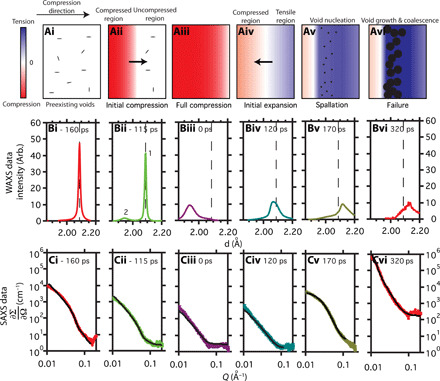
Evolution of scattering data as a shock wave propagates through a Cu foil and illustrative cartoon to aid interpretation. (**ai** to **avi**) From left to right: Sequential illustrations of the longitudinal strain profile and corresponding void distribution in the Cu foils, describing the shock loading cycle to failure as a shock wave propagates through the foil. (**bi** to **bvi**) From left to right: Azimuthally averaged scattering data illustrating the evolution of WAXS as the shock wave propagates through the foil and (**ci** to **cvi**) the corresponding SAXS profiles. The scattering data (bi) to (bvi) and (ci) to (cvi) correspond to the schematic of (ai) to (avi). *t* = 0 ps is defined at full material compression. An increasingly negative time means a shorter lag between the probe and pump, and the initial shock wave has progressed less through the foil. An increasingly positive time corresponds to the rarefaction wave returning through the foil. The peaks labeled 1 and 2 in (bii) correspond to diffraction from the unstrained region ahead of the shock front and strained region behind the shock front, respectively. The dashed vertical lines in (bi) to (bvi) are a reference for the initial {111} peak position. SAXS model fits to data are included as solid black lines (ci to cvi).

Processed and azimuthally averaged scattering data are presented in Fig. 2 (bi to bvi) and (ci to cvi) at six different pump-probe time delays to illustrate the relationship between pump-probe time delay and the evolution in scattering data. The {111} peak at ∼2.086 Å [determined by a Voigt peak fitting routine (*37*)], which corresponds to the material that has yet to be compressed by the shock, is observed to decrease in intensity as time proceeds, whereas a diffraction peak at ∼1.941 Å, associated with the material compressed by the shock, develops with increasing pump-probe time delays (Fig. 2, bi to biii). Eventually, the peak at ∼2.086 Å reaches zero intensity (Fig. 2, biii), which we interpret as the time where all the Cu has been subject to compression, and the shock wave has reached the rear surface of the Cu layer. We define this point to be the time zero reference, *t*_0_. By this definition, an increasingly negative time means a shorter lag between the probe and pump, and the initial shock wave has progressed less through the foil. An increasingly positive time corresponds to the rarefaction wave returning back through the foil.

The SAXS data shown in Fig. 2 (ci to ciii) correspond to the WAXS data of (bi) to (biii). The SAXS intensity decreases, and the scattering profile flattens with increasing time lag between the pump and probe (ci to ciii). It remains flattened (civ) until a time of 170 ps (cv), when the SAXS intensity increases and a change in the scattering profile is observed (cv and cvi). The corresponding WAXS data show the diffraction peak shifting to higher *d*-spacing (biii to bvi).

## DISCUSSION

### SAXS analysis—Initial conditions

The SAXS data collected on the unshocked Cu foils indicate that, owing to the deposition method by which they were made, they already contained voids. The APS SAXS/USAXS instrument records a broader low-angle *Q* range than the LCLS instrument (fig. S1B) and thus can give more information on the initial void distribution, although we stress that the LCLS data are totally consistent with the analysis based on that collected at APS. The azimuthally averaged APS SAXS/USAXS data recorded for the initial condition Cu with substrate samples were iteratively fitted with an oblate spheroid SAXS model by a least-squares refinement. The oblate spheroid model determines a major and minor diameter of approximately 85 and 13 nm, respectively. This shape is a reasonable approximation for grain boundary porosity between grains. The dimensions correspond to the length scales of the grain boundary pore width and length observed in Fig. 1 (C and D); thus, we conclude that the small-angle scattering arises from the scattering length density contrast between the Cu material and voids. Given the void dimensions, grain boundaries that contain these pores are incoherent. The major grain boundary diameter of 85 nm determined by USAXS is in good agreement with the mean grain width of approximately 80 nm determined by image analysis from electron microscopy.

The scattering feature associated with the major dimension lies outside the LCLS low *Q* detector range and could only be observed at the APS USAXS/SAXS instrument. The USAXS low *Q* detector range at the APS beamline is obtained by inserting an advanced-design Bonse-Hart USAXS camera to complement a fixed-length SAXS detector. A Bonse-Hart detector is not suitable for in situ recording of rapid evolution processes and was only used for this initial characterization.

### Direct observation of material response during compression

A two-dimensional simplified cartoon illustrates our results at different stages of shock-induced strain propagation through a foil (Fig. 2, ai to avi), with corresponding WAXS (bi to bvi) and SAXS data (ci to cvi) for each stage of deformation. The cartoon (Fig. 2, ai to avi) illustrates the variation of internal strain as the shock wave propagates through the foil, while the x-ray scattering data (Fig. 2, bi to bvi) are an overall averaged microstructure snapshot through the bulk of the material as deformation occurs. The different stages of shock wave propagation represented in the cartoon that lead to material failure can be categorized as (ai) uncompressed material before shock; (aii) a combination of shock-compressed material behind the shock front and uncompressed material ahead of the shock front, as the shock wave propagates through the sample; (aiii) all the Cu-layer material being compressed when the shock wave reaches the rear surface of that layer; (aiv) relaxation of the compressive strain and subsequent material expansion by a counterpropagating rarefaction wave; (av) a region of tension develops within the foil beyond the yield strength, nucleating voids and representing the onset of failure; (avi) growth and coalescence of the voids causing material fracture.

From the WAXS, we see that, initially, the sample is strain free (Fig. 2, ai) with a {111} peak at 2.086 Å (Fig. 2, bi). This corresponds to a lattice constant of about 3.613 Å, in agreement with literature values (3.6149 Å) (*38*). The preexisting voids discussed above are evident in the LCLS SAXS data, as shown in Fig. 2 (ci). As the shock wave starts to progress through the foil (Fig. 2, aii), a distinct {111} diffraction peak at lower *d*-spacing (∼1.941 Å) develops, which is associated with the compressed region, and grows in intensity as the shock wave passes deeper into the sample. Conversely, the original {111} peak at 2.086 Å (associated with the uncompressed lattice that lies ahead of the shock front) decreases in intensity as the shock wave passes deeper into the sample. This stage of high strain rate deformation is observed in Fig. 2 (bii), where the {111} peaks from the uncompressed and compressed regions are labeled as 1 and 2, respectively, and correspond to a 19% volumetric compression of the material assuming an ideal hydrostatic response. A rapid approach to hydrostatic compression owing to plastic flow is suggested by the lack of a distinct elastic response, as seen in previous experiments (*9*), and is supported by the subsequent MD simulations.

The schematic of Fig. 2 (aii) also illustrates the closure of preexisting pores in the compressed foil region. These voids are closed in the compressed region behind the compression wavefront but unaltered ahead of it. This conclusion is consistent with the subsequent SAXS modeling results and MD simulation. The scattering length density contrast between Cu and vacuum is large (Δρ^2^ ∼ 3630 × 10^20^ cm^−4^ at 8.8 keV); therefore, a small change in void fraction causes a clear intensity change in the SAXS profile. While the material compression is observed by the diffraction data (Fig. 2, bii), the closure of pores in the compressed region and therefore the reduction in pore volume fraction in the material bulk are simultaneously observed by the decrease in SAXS intensity (Fig. 2, cii).

The point when the shock reaches the rear surface of the Cu, and thus all of the Cu sample is under compression, is illustrated in Fig. 2 (aiii) and is identified by the WAXS profile shown in Fig. 2 (biii), where the {111} peak corresponding to the uncompressed Cu lattice is no longer seen in the diffraction pattern, and the {111} peak intensity at lower *d*-spacing corresponding to the compressed Cu lattice is at a maximum. A lower limit on the strain rate of $\overset{\cdot }{\varepsilon }∼0.5\times 10^{9}\;s^{-1}$ is approximated from this change in lattice spacing and time taken to achieve compression of all of the sample.

It can be seen from the SAXS data in Fig. 2 (ciii) that, at this time, the scattering curve has been flattened, and the intensity is at a minimum, indicating full closure of pores, i.e., approaching zero volume fraction. Figure S2 shows additional WAXS and SAXS data to highlight the dynamic nature of the shock propagation process.

### Quantification of void evolution during compression

**Fig. 3:**
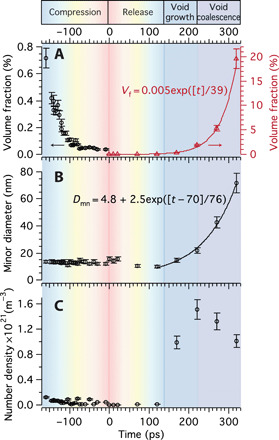
Evolution of microstructure as a shock wave propagates through a Cu foil, deduced from SAXS model fits to experimental data. (**A**) Void volume fraction, (**B**) void minor dimension, and (**C**) void number density with increasing pump-probe delay times, deduced from SAXS model fits to the experimental data. *t*_0_ at 0 ps refers to the time in the shock event at which the sample was fully compressed. Exponential fits to the deduced parameters are included, as well as error bars of 10%, calculated from χ^2^ analyses of the fitting parameters.

The evolution of the microstructural parameters deduced from the SAXS model fits to the data during shock compression are presented in Fig. 3, while model fits to data are presented in Fig. 2 (ci to cvi) and fig. S2 (C and D). The scattering length density contrast during compression arises from the unaltered voids ahead of the shock front. Therefore, it is reasonable to fit the SAXS data during compression with the same oblate spheroid model that was established with the USAXS data from the initial sample condition and assume a constant aspect ratio. The void volume fraction decreases during compression (Fig. 3A), corresponding to the decrease in SAXS intensity observed in Fig. 2 (ci to ciii). The average void size is unchanged during the compression phase, as shown in Fig. 3B, reaffirming that the voids in the compressed region are closed, while those ahead of the wavefront remain unaltered, and that using the same SAXS model for the compression data as the nonshocked measurement is reasonable.

### Direct observation of material response during rarefaction

Following the shock reaching the rear surface of the Cu, a rarefaction waves travel back through the foil releasing the compressive strain, illustrated in Fig. 2 (aiv), and a region of tension develops as the rarefaction wave traveling back from the rear surface interacts with the decaying region of the shock. The lattice response during this expansion is very different to that during compression because of the complex interaction of these two waves; however, the averaged diffraction is still very informative, as shown in Fig. 2 (bi to bvi). In the averaged WAXS data (Fig. 2, biv), the relief of compressive strain is apparent as the {111} peak moves to higher *d*-spacing. At a time of 120 ps, a large region of the foil reaches net tension, observed in the data as a broad peak width of the {111} peak beyond 2.086 Å (Fig. 2, biv). During the release of the compressive strain and development of net tensile strain, the intensity of the SAXS pattern remains flat, up to 120 ps (Fig. 2, civ). This indicates that, up until this time, no region of the foil has reached the threshold strain required for void formation. Again, fig. S2 shows additional WAXS and SAXS data to highlight the dynamic nature of the rarefaction process.

### Spall strength at high strain rate

**Fig. 4:**
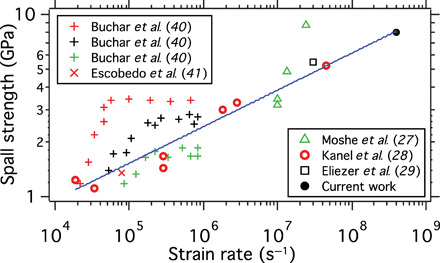
Comparison of experimentally determined high strain rate spall strengths for polycrystalline Cu samples. Buchar *et al.* (*40*) measured three different grain sizes: 94 μm (red data points), 139 μm (black data points), and 185 μm (green data points). Escobedo *et al.* (*41*) also determined the spall strength for samples with average grain size between 30 and 200 μm. The data of Kanel *et al.* (*28*) and the current work are fitted by a straight line on a log-log plot (spall strength = ${\overset{\cdot }{\varepsilon }}^{m}\cdot 10^{b}$, where *m* is the slope and *b* is the intercept point on the log plot).

The tensile strain continues to increase with increasing pump-probe time delays as shown in Fig. 2bv and fig. S2B. The maximum volumetric expansion is approximately 6% based on the shift in the {111} peak position under isotropic expansion. This is reached between 170 and 320 ps (Fig. 2, bv and bvi). This corresponds to a spall strength of approximately 8.5 GPa calculated for a room temperature bulk modulus [140 GPa (*39*)]. To our knowledge, this is the first determination of polycrystal Cu spall strength based on experiment with a strain rate greater than ∼10^7^ s^−1^. Published Cu spall strengths based on experiments at high strain rates are presented alongside the present data in Fig. 4. Buchar *et al.* (*40*) determined spall strengths between 1.2 and 3.4 GPa for strain rates of $10^{4}<\overset{\cdot }{\varepsilon }<10^{6}\;s^{-1}$ in Cu polycrystals with average grain sizes of 94 μm (red data points), 139 μm (black data points), and 185 μm (green data points). They concluded that (i) a critical strain rate existed where the spall strength plateaus and this corresponds to a fracture mode transition from intergranular to intragranular, and (ii) the spall strength is dependent on grain size, with finer grains showing superior spall strength. In contrast, Escobedo *et al.* (*41*) concluded the opposite—that at a strain rate of ε ∼ 0.8 × 10^5^ s^−1^, the spall strength remained ∼1.35 GPa for average grain sizes between 30 and 200 μm. Most recently, Remington *et al.* (*36*) examined the spall strength dependency on grain size and strain rate in Ta in an effort to isolate these two variables. They concluded that the spall strength increases with grain size, between 0.1 and 200 μm at $6\times 10^{6}<\overset{\cdot }{\varepsilon }<5\times 10^{7}\;s^{-1}$.

It is clear from the plotted data (Fig. 4) that the spall strength of Cu is strain rate dependent, as also concluded by Remington *et al.* (*36*) for Ta. Moshe *et al.* (*27*) concluded that the spall strength varies slowly with the strain rate, but a critical phenomenon occurs at strain rates of about 10^7^ s^−1^, observed as a sudden approach to the theoretical value of the spall strength. Kanel *et al.* (*28*) assembled data of Razorenov, Tonks, and Paisley (*42*–*44*) and showed that the data are well fitted by a straight line on a log-log plot (spall strength = ${\overset{\cdot }{\varepsilon }}^{m}\cdot 10^{b}$, where *m* is the slope and *b* is the intercept point on the log plot). In Fig. 4, the same fit to data is performed for the data assembled by Kanel *et al.*, who find a value of *m* ≈ 5, and also including the high strain rate spall strength determined here. It is seen that our measurement of spall strength at the highest strain rate recorded is in agreement to the data presented by Kanel *et al.* (*28*), and not to the theory presented by Moshe *et al.* (*27*). It can be argued that three of the four data points of Moshe *et al.*’s results are fitted by the curve presented here and that the fourth measurement may be outlying data. The spall strength determined by Eliezer *et al.* (*29*) at ε ∼ 3 × 10^7^ s^−1^ is also in good agreement to the fit. Kanel *et al.* (*28*), Eliezer *et al.* (*29*), and Moshe *et al.* (*27*) do not discuss sample grain size, so it is not possible to compare a grain size effect in the experimental data.

### Direct observation of material response during spall

As the material is pulled into tension (see the data in Fig. 2, cv, taken at 170 ps), there is an order of magnitude increase in the SAXS intensity, signifying pore formation, with the associated tensile strain shown in Fig. 2 (bv). This is illustrated in the schematic in Fig. 2 (av). The SAXS intensity continues to increase with increasing pump-probe time delays due to an increase in void volume fraction, and the observed scattering profile shifts to lower *Q* corresponding to void growth, as seen in Fig. 2 (cvi) and fig. S2D. A region of tension has developed beyond the spallation strength, subsequent material deformation requires spallation, and thus a high number density of voids nucleate and grow. This is supported by SAXS modeling and MD simulations presented in the subsequent sections. Complexities such as the strain relief around void growth (*33*, *45*) are not incorporated into the simplified schematic of Fig. 2 (av and avi), nor can such a localized region of strain relief be isolated from the averaged WAXS data where the accumulation of net tensile strain dominates the data.

It was necessary to reduce the beam intensity for measurement times greater than 170 ps to avoid SAXS detector saturation in the low *Q* range. The absolute scattering intensity calibration process includes a rescaling of the measured SAXS data, and the rescaling factor is determined from the glassy carbon standards. Thus, if the incident beam intensity is reduced, then the necessary rescaling factor for absolute scattering data is correspondingly increased by the same ratio, and the final data presented remain as absolute scattering intensity (Fig. 2, cvi, and fig. S2D). The SAXS measurements are composed of x-ray counts scattered from the interaction of the beam with the sample but are also composed of some remaining background noise that is not fully removed by the data correction process. For the absolute scattering calibration, this background noise contribution is also rescaled when a lower incident beam intensity is used. This is why a higher background is observed at high *Q* in Fig. 2 (cvi) and fig. S2D. Any background and change to background are easily accounted for in the SAXS modeling process with a flat background variable.

### Quantification of void evolution during spall

The SAXS measurements recorded during spallation, at long pump-probe time delays, were well fitted by a sphere model, rather than an oblate spheroid model. This is consistent with the subsequent MD simulations. The SAXS model fits to data at long pump-probe time delays are shown in Fig. 2 (cv and cvi) and fig. S2D. Although a sphere model may not be a unique solution, it indicates that the voids formed are more uniform in geometry than before shock and that a sphere model can reasonably approximate the observed scattering in the detector range that arises from voids. It has previously been concluded that as the tensile stress field during spallation is almost entirely isotropic, the voids in ductile materials tend to become almost perfectly spherical (*3*, *31*, *46*). However, the opposite has also been suggested by Remington *et al.* (*36*) for nanocrystalline Ta—that spall-induced voids would have an oblate spheroid shape as spall is primarily intergranular in nanocrystalline Ta samples.

An exponential relationship is observed between the shock propagation and void volume fraction (Fig. 3A) and with void size (Fig. 3B) during damage accumulation. The void volume fraction increases to 0.4% between 120 and 170 ps, ∼1.6% at 220 ps, ∼5.3% at 270 ps, and ∼19.5% at 320 ps. For comparison, Johnson (*46*) presented the measured microporosity in Cu through the sample thickness with a maximum of 30% measured on the spall fracture plane. The measurements presented here are at a greater strain rate and finer grain size and averaged through the foil thickness.

Between 120 and 220 ps, the number density of voids increases to a maximum value of ∼1.5 × 10^21^ m^−3^ (Fig. 3C) compared to ∼5 × 10^20^ m^−3^ initially. The void volume fraction and size both increase rapidly during spallation; therefore, a decrease in void number density at times >220 ps (Fig. 3C) corresponds to coalescence. This is illustrated in the schematic in Fig. 2 (avi), with the corresponding WAXS and SAXS shown in Fig. 2 (bvi and cvi, respectively). This represents the first in situ quantification of all stages of high strain rate damage accumulation by cavitation: nucleation, growth, and coalescence leading to ultimate failure (*32*). Considering the material structure as the sample approaches failure at 320 ps, the void volume fraction is ∼19.5%, the void diameter is ∼72 nm, and the interpore distance is ∼100 nm. Therefore, the foil increasingly resembles a foam-like structure toward the point of failure.

### MD simulation

To understand the microstructural response contributing to the WAXS and SAXS signals, we present results of multimillion-atom MD simulations of ramp-compressed porous copper. Given that a comprehensive simulation is not possible, considering the wide range of relevant temporal and spatial scales, a simplified simulation was performed to provide a qualitative physical understanding of aspects of the key experimental results. The simulation is further described in Materials and Methods.

A sample of size 21 × 20 × 1000 nm was simulated in the LAMMPS code (*47*) using the Mishin Cu potential (*48*). This potential has been widely used to study shock-compressed material due to its recreation of Hugoniot (*49*). The sample comprised four columnar grains with the ⟨111⟩ oriented along the compression direction, with the grains separated by planar voids. The sample was driven by a variable force piston using a temporal pulse (*9*) with the front of the pulse steepened by clipping the leading edge as per the experiment. A peak stress of 65 GPa with a rise time of 180 ps approximately matched experimental data. The simulated Cu foil was freestanding, and the underlying Si_3_N_4_ substrate was not taken into account. This simplification was motivated by both a lack of suitable Si_3_N_4_ potential (and a Cu-Si_3_N_4_ interaction potential) and the need to moderate computational cost. However, as for the pressures of interest, the shock impedance of Si_3_N_4_ (*50*) is lower than that of Cu (*51*), and the Cu layer will undergo release when the compression wave reaches the interface, but the degree of release (and hence rate of tensile strain) will be somewhat overestimated by this approximation of ignoring the Si_3_N_4_ substrate.

**Fig. 5:**
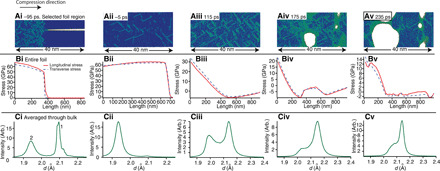
MD simulated evolution of microstructure, internal stresses, and scattering profiles as a shock wave propagates through a Cu foil. (**ai** to **av**) Representative snapshot images of selected foil regions (i.e., not the full foil) for the MD simulation foils at five sequential times of shock wave propagation, illustrating (i) initial compression, (ii) full compression, (iii) expansion, (iv) void nucleation, and (v) void coalescence. The closure of a grain boundary void (white) is shown in (ai), and void nucleation and expansion are also shown in (aiv) and (av). (**bi** to **bv**) The corresponding simulated internal longitudinal stress along the shock direction and the transverse stress averaged for the two normal directions along the full simulated foil, with a positive stress corresponding to compression. (**ci** to **cv**) The corresponding predicted WAXS profiles through the foil bulk. Peaks labeled 1 and 2 of (ci) correspond to scattering arising from the uncompressed and compressed regions, respectively.

Representative selective region snapshots of the simulated microstructure during deformation (from the MD simulations) are shown in Fig. 5 (ai to av), and the color coding is by centrosymmetry parameter (*52*). Corresponding averaged longitudinal stress in the direction of compression and the averaged transverse stress normal to the compression direction through the full simulated sample are shown in Fig. 5 (bi to bv). Corresponding simulated WAXS profiles through the bulk are shown in Fig. 5 (ci to cv). The WAXS profiles are generated using a discrete Fourier transform over the atomic positions (*53*). Note that the relatively narrow cross section of the simulation prohibits meaningful generation of SAXS data.

For the simulated results, the time zero is again defined to be when the compression wave reaches the rear surface so that the results can be compared with the experimental data presented in Fig. 2. The compression phase of the simulation lasts 205 ps, whereupon the compression wave reaches the rear surface. During this time, the longitudinal voids seen in Fig. 5 (ai) are closed by the passage of the compression wave as material relaxes transverse to the loading direction (Fig. 5, aii). A combination of this relaxation and subsequent plastic flow leads to a near hydrostatic compression pathway, with just 1.5 GPa of strength observed at peak compression. The predicted WAXS signal illustrates the development of the (111) peak at 1.94 Å arising from the compressed region and labeled peak 2 in Fig. 5 (ci), in agreement with the experimental data and initial interpretation. The predicted release of compressive strain differs from the observed experimental data (Fig. 5, biv), and this is expected; as discussed above, the influence of the Cu-Si_3_N_4_ interaction was not taken into consideration. The model predicts that a region of tension is developed more rapidly than experimentally observed, within 30 ps following full compression. This region of tension is shown in the predicted averaged longitudinal and transverse stresses in Fig. 5 (biii) and the corresponding WAXS data (Fig. 5, ciii) with a peak position greater than 2.09 Å, and is generated due to a sequence of the decreasing pressure applied at the piston leading to lower stress states and later, more profoundly, as release starts due to wave interaction with the rear surface.

Figure 5 (aiv) highlights that voids are predicted to open within the material between 150 and 190 ps after rear surface release has initiated, driving void fraction to 2.5%. As discussed above, this growth in porosity is somewhat faster than observed in experiment because of the absence of a tamper in the simulations leading to high-amplitude (and rate) release wave formation. These voids are observed to form throughout a large fraction of the material, originating from an ideal crystal sample. It should be noted that the coherent nature of the grain boundaries formed by closure of the initial planar voids and the lack of resolved tensile stress across these boundaries mean that we expect no preferential intergrain void nucleation in these simulations. The voids in this initial void growth phase are approximately spheroidal with eccentricities of 0.4 ± 0.3. The lack of elongation can be understood by noting the highly disordered environment surrounding the voids caused by the appreciable elastic energy released upon rapid void formation. This negates the usual elastic and plastic anisotropy seen in copper leading to near isotropic growth. This supports the conclusion from SAXS modeling that the voids generated during spallation are quite uniform in geometry. From 190 ps onward, voids coalesce, evidenced as an increasing pore fraction with decreased void number. Voids on the verge of coalescence are shown in Fig. 5 (av). Although void growth and increased void fraction are observed to continue beyond 250 ps, wave interactions with the artificially rigid piston lead to a pathological recompression, limiting the time scale over which void growth can be quantitatively studied.

Toward the end of the simulation, a modest tensile strain of 2.5% is inferred from the WAXS pattern. At this stage, in the simulation, the sample is experiencing a range of states. Within the failed material region toward the middle of the sample, stress is close to fully relaxed (<1 GPa), but the sample is partly molten because of the energy released during void formation. This region has an average temperature of 1250 K, close to the melt temperature of 1350 K. Nearer the piston, where sample has not failed by void formation, the sample is slightly cooler, with temperatures ranging from 1100 up to 1250 K, where mixed phase solid and disordered regions are observed. Again, these complexities are not incorporated into the simplified cartoon of Fig. 2 (ai to vi). Note that in both the experimental and simulated WAXS data, the material is seen to return not to its ambient lattice spacing of 2.09 Å but to a value of around 2.09 Å, which is consistent with thermal expansion of the material at around 1300 K.

### Summary

In situ ultrafast-SAXS has enabled direct quantitative determination of material failure during spallation in the sequence of void nucleation, growth, coalescence, and ultimate failure. The elastic strain state was simultaneously determined from Bragg diffraction. The experimental time scales are directly comparable to those that are achievable within MD simulations. The experimentally deduced spall strength is compared with values found in the literature and agrees well with the power-law relationship between spall strength and strain rate proposed by Kanel *et al.* (*28*); however, the effect of grain size is not accounted for in the work presented throughout much of the literature. The voids that nucleate and grow at the onset of failure are determined to be spheroidal, and this is supported with MD simulations.

The development and application of in situ ultrafast-SAXS with femtosecond resolution to quantitatively characterize high strain rate spallation failure, complementing WAXS, is of significance to multiple fields. This work demonstrates a broadening in the range of science that can be performed at XFEL sources and offers the first quantitative determination of the dynamic high strain rate failure process.

## MATERIALS AND METHODS

### Experimental design

LCLS has previously been used to probe crystallographic evolution during high strain rate material compression with a WAXS detector (*9*, *11*). The aim of the current experiment was to quantitatively determine the role of voids throughout the high strain rate shock process at the LCLS with a SAXS detector while simultaneously collecting crystallographic data in the WAXS detector. Sputter-deposited, {111}-oriented polycrystalline Cu samples were chosen to give stronger diffraction peaks in the WAXS detector, and the lattice strain response of Cu at high strain rates is relatively well understood (*9*).

This was a first-of-kind SAXS experiment performed at the LCLS. Therefore, it was particularly important to validate the beamline by measuring known glassy carbon standards and by also comparing SAXS measurements of the Cu samples to those of a calibrated synchrotron beamline (beamline 9-ID at the APS). Complementary microscopy and materials modeling were performed to validate the in situ scattering measurements and to conclude the high strain rate failure mode.

### Electron microscopy

One-micrometer-thick polycrystalline Cu films were vacuum-deposited on <100>-oriented 10-μm-thick Si_3_N_4_ amorphous substrates. Sample texture was measured using EBSD on an Oxford Instruments HKL Nordlys detector with an accelerating voltage of 20 kV. A 50-nm step size was used for the data acquisition, and the resulting pole figures were plotted using the MATLAB Toolbox MTEX. The EBSD confirmed a (111) grain texture in the sample growth direction (Fig. 1B). Secondary electron imaging was conducted using a Zeiss Sigma 300 FESEM operating at an accelerating voltage of 5 kV, and ImageJ software was used for image analysis. The mean grain width was calculated from traced areas using square equivalence from approximately 200 grains.

### Ex situ SAXS

While in situ WAXS measurements during shock at the LCLS Coherent X-ray Imaging (CXI) instrument are now routine [e.g., (*9*)], the SAXS measurements required the development of procedure and validation for the beamline. SAXS measurements of glassy carbon standards (*54*, *55*) were measured on the CXI SAXS/WAXS instrument at LCLS (*56*) with 8.8-keV x-ray energy (fig. S1A).

The Cu deposited films on substrates were characterized on both the LCLS instrument with 8.8-keV x-ray energy and the USAXS instrument at APS with 21-keV x-rays (fig. S1B) (*57*, *58*). The APS USAXS data were processed with the instrument software package Indra (*57*), correcting for sample transmissions, backgrounds, and dark current background in the detectors, and calibrated to absolute scattering intensities with glassy carbon standards (*54*, *55*). A measurement of just the substrate was used for the background measurement. The LCLS raw SAXS data were processed in a similar manner to the USAXS data, using the Nika software package (*59*), with additional corrections for dead pixels.

### Ex situ SAXS modeling

From the microscopy (Fig. 1C), we conclude that SAXS arises from the scattering contrast between Cu and internal nanoscale voids present at grain boundaries. The USAXS data of the unshocked sample were fitted by an oblate spheroid using a least-squares refinement with volume fraction, spheroid size, and a flat background as fitting variables. The fit is shown in fig. S1B. The scattering length density contrast Δρ^2^ between Cu and air is ∼3630 × 10^20^ cm^−4^ (*60*). The determined volume fraction is essentially independent of the SAXS model implemented.

SAXS modeling requires simplifying assumptions, and in this case, full three-dimensional averaging is included in the model. A more complicated SAXS model could, in principle, be developed to account for void and grain anisotropy; however, such a model would require model fitting to scattering data from multiple foil orientations, which was not feasible given the beam time available, and thus for simplicity, and given that an oblate spheroid morphology and the microstructural parameters deduced both agree reasonably well with the microscopy, a three-dimensional averaged model was deemed satisfactory.

### Pump-probe method

In situ simultaneous SAXS/WAXS measurements during high strain rate deformation were conducted on the LCLS instrument as shown in Fig. 1. Arrays of sputtered Cu-with-substrate samples were placed on cassettes, allowing sequential mounting. Shocks were driven through the Cu surface along the <111> directions via laser ablation of each Cu with substrate sample using 17-mJ output from the CXI Ti:sapphire picosecond laser. The samples were simultaneously probed by 8.8-keV XFEL pulses with bandwidth Δ*E*/*E* = 0.2 to 0.5%, 30-fs pulse length, and focused to a 20-μm spot aligned to the center of the driven region where the laser uniformity is maximized. Scattering data were simultaneously recorded in the front detector (WAXS) and rear detector (SAXS). The pump-probe time delay was increased for each sequential measurement, producing a sequence of femtosecond snapshots at pump-probe time delay intervals of 5 to 10 ps (corresponding to an incremental shock propagation of 20 to 55 nm) during material compression and 50 ps (275 nm) during material expansion. A number of repeated measurements at the same pump-probe time delays confirmed that the scattering was consistent from sample to sample. The x-ray intensity was decreased at long pump-probe time delays to avoid SAXS detector saturation. In total, measurements were taken at 35 different pump-probe time delays within a 480-ps range. Pulses were focused onto the sample with a spatially Gaussian profile with 260-μm 1/*e*^2^ diameter and a temporally clipped leading edge to ensure a sharper shock rise time and thus a higher strain rate release.

Note that the sample is impinged upon directly by the laser, rather than via an intermediate ablation layer. Given the laser intensity *I* < 10^12^ W cm^−2^, we expect mass ablation rates of less than 10^5^ g cm^−2^ s and thus minimal loss of material to ablation of order 10 nm (*61*). There is also no clear evidence of extensive loss of sample thickness through ablation in the SAXS data. The SAXS intensity remains constant for a period of time during full compression, and this could not be the case if a considerable loss of sample thickness was occurring. In addition, despite copper’s relatively high thermal diffusivity of 1.1 cm^2^ s^−1^ (*62*), conduction of heat through the full foil thickness would take approximately 9 ns, an order of magnitude longer than the time scale of the experiment.

### In situ SAXS modeling

The SAXS data during compression were fitted with the same oblate spheroid model that was established for the initial sample condition (ex situ measurement), and a constant aspect ratio was assumed. This is reasonable, as the scattering length density contrast during compression is arising from the unaltered voids ahead of the shock front.

At full compression (0 ps), the low-intensity scattering observed at low *Q* and the flat background at high *Q* are well fitted by the spheroid model. Because of the large scattering length density contrast between the Cu material and voids, the model fit to data with a pore volume fraction of just 0.01% describes the observed profile (Fig. 2, ciii). This is termed the fully compressed state with full closure of pores.

The SAXS measurements recorded during spallation, at long pump-probe time delays, were well fitted by a sphere model, and not by an oblate spheroid model. Model fits to data are shown in Fig. 2 (ci to cvi).

### MD simulation

A sample of size 21 × 20 × 1000 nm was simulated in the LAMMPS code (*47*) using the Mishin EAM1 potential (*48*), with periodic boundaries applied along *x* and *y*. As with the experimental samples, [111] was oriented along *z*, the compression direction, with $\left[1\bar{1}0\right]$ along *x* and $\left[11\bar{2}\right]$ along *y*. This sample comprised four columnar grains, each running the full length of the sample along *z* (excluding the piston region, which was full density), separated by planar voids in the *xz* and *yz* planes with widths of 1 supercell (5.1 Å in *x* and 8.9 Å in *y*). This leads to a total number of simulated atoms of 30,660,080.

The sample was equilibrated to 300 K for 5 ps under an NVE integrator, where the NVE ensemble refers to the number of particles in the system (symbol: N), the system’s volume (symbol: V), as well as the total energy in the system (symbol: E). This also allows relaxation at the void surfaces, which leads to a porosity before compression of 9%, somewhat above the ∼1% in the experimental samples.

The sample was driven by a variable force piston using a temporal pulse related to that assumed by Milathianaki *et al.* (*9*), where longitudinal drive stress as a function of time, *t*, is given by σ(*t*) ∝ *t*^2^*e*^−γ*t*^. A peak stress of 65 GPa with γ chosen to achieve a rise time to maximum pressure of 180 ps was used to approximately match experimental data. The front of this pulse was steepened by cropping the first 50 ps of the leading edge, leading to an initial shock of pressure 23 GPa, below the elastic limit of Cu under similar loading conditions in the absence of voids (*9*, *63*). No constraint is applied at the rear surface of the sample, corresponding to a freestanding foil.

The simulation was allowed to run for 420 ps, at which point the release wave reflects from the piston, leading to a nonphysical recompression of the sample, and closure of voids. Void regions were monitored by mapping atoms into a discrete grid of cell size 5 Å and defining void region to be any cell with zero atoms to be within a void region. This leads to an underestimate of void size and porosity in smaller voids (dimension, ∼5Å) and sets a minimum size of void, which can be detected. However, for the voids seen on release of the sample, typically growing to dimensions of more than 50 Å within 20 ps, this is a relatively small (∼5%) error.

Simulated scattering was calculated by taking discrete samples of reciprocal space intensity via a Fourier transform (*53*). The intensity is summed around spherical shells, each corresponding to a unique momentum transfer, $q=\frac{2\pi }{d}$, where *d* is the spacing of the diffracting planes. Samples were quasi-uniformly distributed over the sphere surface, and only those corresponding to a scattering angle 65^∘^ ≤ 2θ ≤ 75^∘^ are included in the sum, which corresponds to a scattering geometry equivalent to the experimental WAXS signal from the subset of {111} planes orientated appropriately to meet the Bragg condition.

## Supplementary Material

http://advances.sciencemag.org/cgi/content/full/6/51/eabb4434/DC1

Adobe PDF - abb4434_SM.pdf

Femtosecond quantification of void evolution during rapid material failure

## SUPPLEMENTARY MATERIALS

Supplementary material for this article is available at http://advances.sciencemag.org/cgi/content/full/6/51/eabb4434/DC1

## References

[1] Tuler F. R., Butcher B. M., A criterion for the time dependence of dynamic fracture. Int. J. Fract. Mech. 4, 431–437 (1968).

[2] Seaman L., Curran D. R., Shockey D. A., Computational models for ductile and brittle fracture. J. Appl. Phys. 47, 4814–4826 (1976).

[3] A. K. Zurek, *Dynamic Ductile Evolution and Tensile Fracture: New Experimental Insights for Models Evaluation* (Elsevier Science, 2000), pp. 125–134.

[4] Johnson Q., Mitchell A., Evans L., X-ray diffraction evidence for crystalline order and isotropic compression during the shock-wave process. Nature 231, 310–311 (1971).

[5] Wark J. S., Whitlock R. R., Hauer A., Swain J. E., Solone P. J., Shock launching in silicon studied with use of pulsed x-ray diffraction. Phys. Rev. B 35, 9391–9394 (1987). 10.1103/physrevb.35.9391 9941362

[6] Wark J. S., Whitlock R. R., Hauer A. A., Swain J. E., Solone P. J., Subnanosecond x-ray diffraction from laser-shocked crystals. Phys. Rev. B 40, 5705–5714 (1989). 10.1103/physrevb.40.5705 9992608

[7] Kalantar D. H., Belak J. F., Collins G. W., Colvin J. D., Davies H. M., Eggert J. H., Germann T. C., Hawreliak J., Holian B. L., Kadau K., Lomdahl P. S., Lorenzana H. E., Meyers M. A., Rosolankova K., Schneider M. S., Sheppard J., Stölken J. S., Wark J. S., Direct observation of theα−εTransition in shock-compressed iron via nanosecond x-ray diffraction. Phys. Rev. Lett. 95, 075502 (2005). 16196791 10.1103/PhysRevLett.95.075502

[8] Turneaure S. J., Gupta Y. M., Zimmerman K., Perkins K., Yoo C. S., Shen G., Real-time microstructure of shocked LiF crystals: Use of synchrotron x-rays. J. Appl. Phys. 105, 053520 (2009).

[9] Milathianaki D., Boutet S., Williams G. J., Higginbotham A., Ratner D., Gleason A. E., Messerschmidt M., Seibert M. M., Swift D. C., Hering P., Robinson J., White W. E., Wark J. S., Femtosecond visualization of lattice dynamics in shock-compressed matter. Science 342, 220–223 (2013).24115435

[10] Swinburne T. D., Glavicic M. G., Rahman K. M., Jones N. G., Coakley J., Eakins D. E., White T. G., Tong V., Milathianaki D., Williams G. J., Rugg D., Sutton A. P., Dye D., Picosecond dynamics of a shock-driven displacive phase transformation in zr. Phys. Rev. B 93, 144119 (2016).

[11] Wehrenberg C. E., McGonegle D., Bolme C., Higginbotham A., Lazicki A., Lee H. J., Nagler B., Park H.-S., Remington B. A., Rudd R. E., Sliwa M., Suggit M., Swift D., Tavella F., Zepeda-Ruiz L., Wark J. S., *In situ* x-ray diffraction measurement of shock-wave-driven twinning and lattice dynamics. Nature 550, 496–499 (2017). 29072261 10.1038/nature24061

[12] Wark J. S., Woolsey N. C., Whitlock R. R., Novel measurements of high-dynamic crystal strength by picosecond x-ray diffraction. Appl. Phys. Lett. 61, 651–653 (1992).

[13] Higginbotham A., Stubley P. G., Comley A. J., Eggert J. H., Foster J. M., Kalantar D. H., McGonegle D., Patel S., Peacock L. J., Rothman S. D., Smith R. F., Suggit M. J., Wark J. S., Inelastic response of silicon to shock compression. Sci. Rep. 6, 24211 (2016). 27071341 10.1038/srep24211 PMC4829838

[14] Emma P., Akre R., Arthur J., Bionta R., Bostedt C., Bozek J., Brachmann A., Bucksbaum P., Coffee R., Decker F.-J., Ding Y., Dowell D., Edstrom S., Fisher A., Frisch J., Gilevich S., Hastings J., Hays G., Hering P., Huang Z., Iverson R., Loos H., Messerschmidt M., Miahnahri A., Moeller S., Nuhn H.-D., Pile G., Ratner D., Rzepiela J., Schultz D., Smith T., Stefan P., Tompkins H., Turner J., Welch J., White W., Wu J., Yocky G., Galayda J., First lasing and operation of an ångstrom-wavelength free-electron laser. Nat. Photonics 4, 641–647 (2010).

[15] P. Hart, S. Boutet, G. Carini, M. Dubrovin, B. Duda, D. Fritz, G. Haller, R. Herbst, S. Herrmann, C. Kenney, N. Kurita, H. Lemke, M. Messerschmidt, M. Nordby, J. Pines, D. Schafer, M. Swift, M. Weaver, G. Williams, D. Zhu, N. V. Bakel, J. Morse, in *X-Ray Free-Electron Lasers: Beam Diagnostics, Beamline Instrumentation, and Applications,* S. P. Moeller, M. Yabashi, S. P. Hau-Riege, Eds. (SPIE, 2012), vol. 8504, pp. 51–61.

[16] Shiryaev A., Dembo K., Klyuev Y., Naletov A., Feigelson B., Small-angle x-ray scattering of extended defects in diamonds. J. Appl. Crystallogr. 36, 420–424 (2003).

[17] Gilbert J. A., Kropf A. J., Kariuki N. N., De Crane S., Wang X., Rasouli S., Yu K., Ferreira P. J., Morgan D., Myers D. J., In-operando anomalous small-angle x-ray scattering investigation of Pt_3_Co catalyst degradation in aqueous and fuel cell environments. J. Electrochem. Soc. 162, F1487–F1497 (2015).

[18] Coakley J., Vorontsov V. A., Jones N. G., Radecka A., Bagot P. A. J., Littrell K. C., Heenan R. K., Hu F., Magyar A. P., Bell D. C., Dye D., Precipitation processes in the beta-titanium alloy Ti–5Al–5Mo–5V–3Cr. J. Alloys Compd. 646, 946–953 (2015).

[19] Koch M. H. J., Vachette P., Svergun D. I., Small-angle scattering: A view on the properties, structures and structural changes of biological macromolecules in solution. Q. Rev. Biophys. 36, 147–227 (2003). 14686102 10.1017/s0033583503003871

[20] Allen A. J., Characterization of ceramics by x-ray and neutron small-angle scattering. J. Am. Ceram. Soc. 88, 1367–1381 (2005).

[21] Jafta C. J., Petzold A., Risse S., Clemens D., Wallacher D., Goerigk G., Ballauff M., Correlating pore size and shape to local disorder in microporous carbon: A combined small angle neutron and x-ray scattering study. Carbon 123, 440–447 (2017).

[22] Peterlik H., Fratzl P., Kromp K., Pore structure of carbon/carbon composites studied by small-angle x-ray scattering. Carbon 32, 939–945 (1994).

[23] Coppola R., Klimenkov M., Lindau R., Möslang A., Rieth M., Valli M., Radiation damage studies in fusion reactor steels by means of small-angle neutron scattering (SANS). Phys. B Condens. Matter 551, 407–412 (2018).

[24] Kluge T., Rodel C., Rodel M., Pelka A., McBride E. E., Fletcher L. B., Harmand M., Krygier A., Higginbotham A., Bussmann M., Galtier E., Gamboa E., Garcia A. L., Garten M., Glenzer S. H., Granados E., Gutt C., Lee H. J., Nagler B., Schumaker W., Tavella F., Zacharias M., Schramm U., Cowan T. E., Nanometer-scale characterization of laser-driven compression, shocks, and phase transitions, by x-ray scattering using free electron lasers. Phys. Plasmas 24, 102709 (2017).

[25] Firestone M. A., Dattelbaum D. M., Podlesak D. W., Gustavsen R. L., Huber R. C., Ringstrand B. S., Watkins E. B., Jensen B. L., Willey T., Lauderbach L., Hodgin R., Bagge-Hansen M., van Buuren A., Seifert S., Graber T., Structural evolution of detonation carbon in composition-B by x-ray scattering. AIP Conf. Proc. 1793, 030010 (2017).

[26] Gustavsen R. L., Dattelbaum D. M., Watkins E. B., Firestone M. A., Podlesak D. W., Jensen B. J., Ringstrand B. S., Huber R. C., Mang J. T., Johnson C. E., Velizhanin K. A., Willey T. M., Hansen D. W., May C. M., Hodgin R. L., Bagge-Hansen M., van Buuren A. W., Lauderbach L. M., Jones A. C., Graber T. J., Sinclair N., Seifert S., Gog T., Time resolved small angle x-ray scattering experiments performed on detonating explosives at the advanced photon source: Calculation of the time and distance between the detonation front and the x-ray beam. J. Appl. Phys. 121, 105902 (2017).

[27] Moshe E., Eliezer S., Dekel E., Ludmirsky A., Henis Z., Werdiger M., Goldberg I. B., Eliaz N., Eliezer D., An increase of the spall strength in aluminum, copper, and Metglas at strain rates larger than 107 s^−1^. J. Appl. Phys. 83, 4004–4011 (1998).

[28] Kanel G. I., Fortov V. E., Razorenov S. V., Shock waves in condensed-state physics. Phys. Uspekhi 50, 771 (2007).

[29] Eliezer S., Gilath I., Bar-Noy T., Laser induced spall in metals: Experiment and simulation. J. Appl. Phys. 67, 715–724 (1990).

[30] J. S. Rinehart, *Historical Perspective: Metallurgical Effects of High Strain Rate Deformation and Fabrication* (Springer US, 1981), pp. 3–20.

[31] Curran D., Seaman L., Shockey D., Dynamic failure of solids. Phys. Rep. 147, 253–388 (1987).

[32] Meyers M. A., Aimone C. T., Dynamic fracture (spalling) of metals. Prog. Mater. Sci. 28, 1–96 (1983).

[33] Belak J., On the nucleation and growth of voids at high strain rates. J. Computer-Aided Mater. Des. 5, 193–206 (1998).

[34] Hahn E. N., Germann T. C., Ravelo R., Hammerberg J. E., Meyers M. A., On the ultimate tensile strength of tantalum. Acta Mater. 126, 313–328 (2017).

[35] Luo S.-N., An Q., Germann T. C., Han L.-B., Shock-induced spall in solid and liquid cu at extreme strain rates. J. Appl. Phys. 106, 013502 (2009).

[36] Remington T., Hahn E., Zhao S., Flanagan R., Mertens J., Sabbaghianrad S., Langdon T., Wehrenberg C., Maddox B., Swift D., Remington B., Chawla N., Meyers M., Spall strength dependence on grain size and strain rate in tantalum. Acta Mater. 158, 313–329 (2018).

[37] Dye D., Coakley J., Vorontsov V., Stone H., Rogge R., Elastic moduli and load partitioning in a single-crystal nickel superalloy. Scr. Mater. 61, 109–112 (2009).

[38] Straumanis M. E., Yu L. S., Lattice parameters, densities, expansion coefficients and perfection of structure of Cu and of Cu–In α phase. Acta Crystallogr. A 25, 676–682 (1969).

[39] Gathers G. R., Dynamic methods for investigating thermophysical properties of matter at very high temperatures and pressures. Rep. Prog. Phys. 49, 341–396 (1986).

[40] Buchar J., Elices M., Cortez R., The influence of grain size on the spall fracture of copper. J. Phys. IV France 01, C3-623–C3-630 (1991).

[41] Escobedo J. P., Dennis-Koller D., Cerreta E. K., Patterson B. M., Bronkhorst C. A., Hansen B. L., Tonks D., Lebensohn R. A., Effects of grain size and boundary structure on the dynamic tensile response of copper. J. Appl. Phys. 110, 033513 (2011).

[42] G. I. Kanel, S. V. Razorenov, V. E. Fortov, *Shock Wave Phenomena in Condensed Media* (Yanus-K, 1996).

[43] Tonks D. L., Alexander D. J., Sheffield S. A., Robbins D. L., Zurek A. K., Thissell W. R., Spallation strength of single crystal and polycrystalline copper. J. Phys. IV France 10, Pr9-787–Pr9-792 (2000).

[44] D. L. Paisley, R. H. Warnes, R. A. Kopp, Laser-driven flat plate impacts to 100 GPa with sub-nanosecond pulse duration and resolution for material property studies, in *Shock Compression of Condensed Matter*, S. Schmidt, R. Dick, J. Forbes, D. Tasker, Eds. (Elsevier, 1992), pp. 825–828.

[45] Luo S.-N., Germann T. C., Desai T. G., Tonks D. L., An Q., Anisotropic shock response of columnar nanocrystalline cu. J. Appl. Phys. 107, 123507 (2010).

[46] Johnson J. N., Dynamic fracture and spallation in ductile solids. J. Appl. Phys. 52, 2812–2825 (1981).

[47] Plimpton S., Fast parallel algorithms for short-range molecular dynamics. J. Comput. Phys. 117, 1–19 (1995).

[48] Mishin Y., Mehl M. J., Papaconstantopoulos D. A., Voter A. F., Kress J. D., Structural stability and lattice defects in copper: Ab initio, tight-binding, and embedded-atom calculations. Phys. Rev. B Condens. Matter 63, 224106 (2001).

[49] Bringa E. M., Cazamias J. U., Erhart P., Stölken J., Tanushev N., Wirth B. D., Rudd R. E., Caturla M. J., Atomistic shock Hugoniot simulation of single-crystal copper. J. Appl. Phys. 96, 3793–3799 (2004).

[50] Bakanova A. A., Bugaeva V. A., Dudoladov I. P., Trunin R. F., Udarnaya szhimaemost’ nitridov i karbidov mettalov. Izv. Akad. Nauk. SSSR Fiz. Zemli 6, 58–63 (1995).

[51] Walsh J. M., Rice M. H., McQueen R. G., Yarger F. L., Shock-wave compressions of twenty-seven metals. equations of state of metals. Phys. Rev. 108, 196–216 (1957).

[52] Kelchner C. L., Plimpton S. J., Hamilton J. C., Dislocation nucleation and defect structure during surface indentation. Phys. Rev. B 58, 11085–11088 (1998).

[53] Kimminau G., Nagler B., Higginbotham A., Murphy W. J., Park N., Hawreliak J., Kadau K., Germann T. C., Bringa E. M., Kalantar D. H., Lorenzana H. E., Remington B. A., Wark J. S., Simulating picosecond x-ray diffraction from shocked crystals using post-processing molecular dynamics calculations. J. Phys. Condens. Matter 20, 505203 (2008).

[54] Allen A. J., Zhang F., Kline R. J., Guthrie W. F., Ilavsky J., NIST standard reference material 3600: Absolute intensity calibration standard for small-angle X-ray scattering. J. Appl. Crystallogr. 50, 462–474 (2017). 28381972 10.1107/S1600576717001972 PMC5377342

[55] Zhang F., Ilavsky J., Long G. G., Quintana J. P. G., Allen A. J., Jemian P. R., Glassy carbon as an absolute intensity calibration standard for small-angle scattering. Metall. Mater. Trans. A 41, 1151–1158 (2010).

[56] Liang M., Williams G. J., Messerschmidt M., Seibert M. M., Montanez P. A., Hayes M., Milathianaki D., Aquila A., Hunter M. S., Koglin J. E., Schafer D. W., Guillet S., Busse A., Bergan R., Olson W., Fox K., Stewart N., Curtis R., Miahnahri A. A., Boutet S., The Coherent X-ray Imaging instrument at the Linac Coherent Light Source. J. Synchrotron Radiat. 22, 514–519 (2015). 25931062 10.1107/S160057751500449X PMC4416669

[57] Ilavsky J., Jemian P. R., Allen A. J., Zhang F., Levine L. E., Long G. G., Ultra-small-angle X-ray scattering at the Advanced Photon Source. J. Appl. Crystallogr. 42, 469–479 (2009).

[58] Freelon B., Suthar K., Ilavsky J., A multi-length-scale USAXS/SAXS facility: 10–50 keV small-angle X-ray scattering instrument. J. Appl. Crystallogr. 46, 1508–1512 (2013).

[59] Ilavsky J., *Nika*: Software for two-dimensional data reduction. J. Appl. Crystallogr. 45, 324–328 (2012).

[60] Ilavsky J., Jemian P. R., *Irena*: Tool suite for modeling and analysis of small-angle scattering. J. Appl. Crystallogr. 42, 347–353 (2009).

[61] Goldsack T. J., Kilkenny J. D., Rumsby P. T., Determination of mass ablation rates and ablation pressures on spherical targets by ion emission. J. Phys. D. Appl. Phys. 14, L47–L50 (1981).

[62] Parker W. J., Jenkins R. J., Butler C. P., Abbott G. L., Flash method of determining thermal diffusivity, heat capacity, and thermal conductivity. J. Appl. Phys. 32, 1679–1684 (1961).

[63] Dupont V., Germann T. C., Strain rate and orientation dependencies of the strength of single crystalline copper under compression. Phys. Rev. B Condens. Matter 86, 134111 (2012).

