# Supplementary material for: Femtosecond quantification of void evolution during rapid material failure
Source: Sci Adv. 2020 Dec 16;6(51):eabb4434. doi: 10.1126/sciadv.abb4434 (PMC7744076; doi:10.1126/sciadv.abb4434)
Supplement: http://advances.sciencemag.org/cgi/content/full/6/51/eabb4434/DC1 [file supp_6_51_eabb4434__index.html]

Science Advances | Science AdvancesAAASSearchScience AdvancesMenu

## Supplementary Materials

# Femtosecond quantification of void evolution during rapid material failure

James Coakley, Andrew Higginbotham, David McGonegle, Jan Ilavsky, Thomas D. Swinburne, Justin S. Wark, Khandaker M. Rahman, Vassili A. Vorontsov, David Dye, Thomas J. Lane, Sébastien Boutet, Jason Koglin, Joseph Robinson, Despina Milathianaki

Download Supplement

**This PDF file includes:**

- Figs. S1 and S2

**Files in this Data Supplement:**

- Adobe PDF - abb4434\_SM.pdf
