# Supplementary material for: Femtosecond quantification of void evolution during rapid material failure
Source: Sci Adv. 2020 Dec 16;6(51):eabb4434. doi: 10.1126/sciadv.abb4434 (PMC7744076; doi:10.1126/sciadv.abb4434)
Supplement: http://advances.sciencemag.org/cgi/content/full/6/51/eabb4434/DC1 [file supp_6_51_eabb4434__1.pdf]

Science Advances
AAAS

advances.sciencemag.org/cgi/content/full/6/51/eabb4434/DC1

Supplementary Materials for

Femtosecond quantification of void evolution during rapid material failure

James Coakley*, Andrew Higginbotham, David McGonegle, Jan Ilavsky, Thomas D. Swinburne, Justin S. Wark, Khandaker M. Rahman, Vassili A. Vorontsov, David Dye, Thomas J. Lane, Sébastien Boutet, Jason Koglin, Joseph Robinson, Despina Milathianaki

*Corresponding author. Email: jcoakley@miami.edu

Published 16 December 2020, *Sci. Adv.* **6**, eabb4434 (2020)
DOI: 10.1126/sciadv.abb4434

This PDF file includes:

Figs. S1 and S2

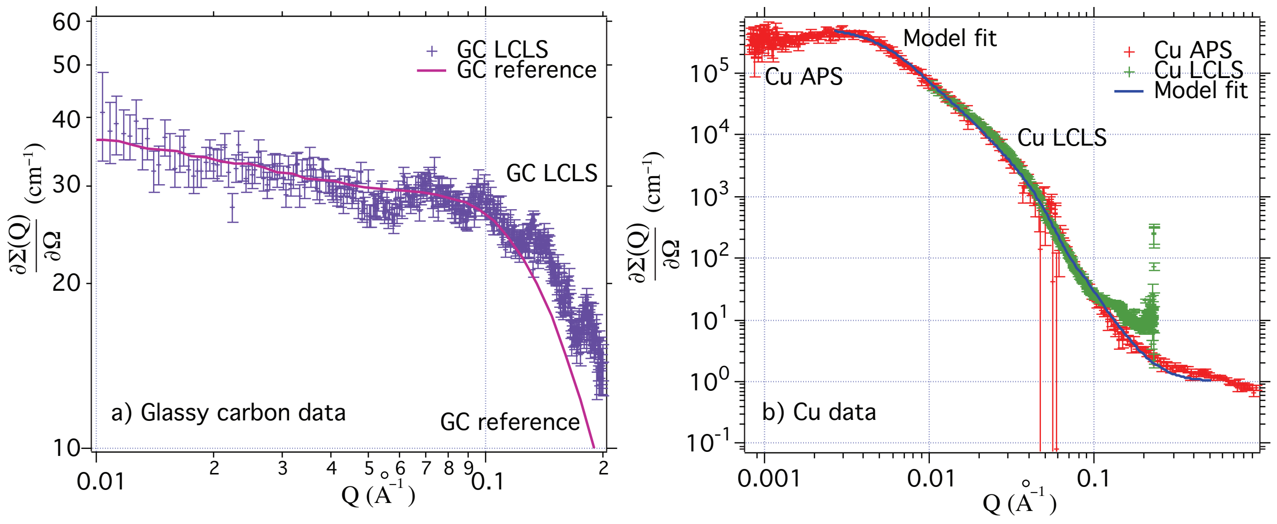

Fig. S. 1: **Comparing fully reduced SAXS data determined at the LCLS to a standard reference measurement and to data determined at the USAXS instrument at APS**. The fully reduced SAXS data from **a)** the glassy carbon (GC) sample measured at LCLS and the standard reference measurement for absolute scattering intensity reduction, **b)** a sputtered Cu sample with substrate measured at the USAXS instrument at APS to a sputtered Cu sample with substrate measured at LCLS. For direct comparison, the LCLS data has been corrected by the difference in the beam energy between the two measurements. The model fit to the APS Cu data is also shown.

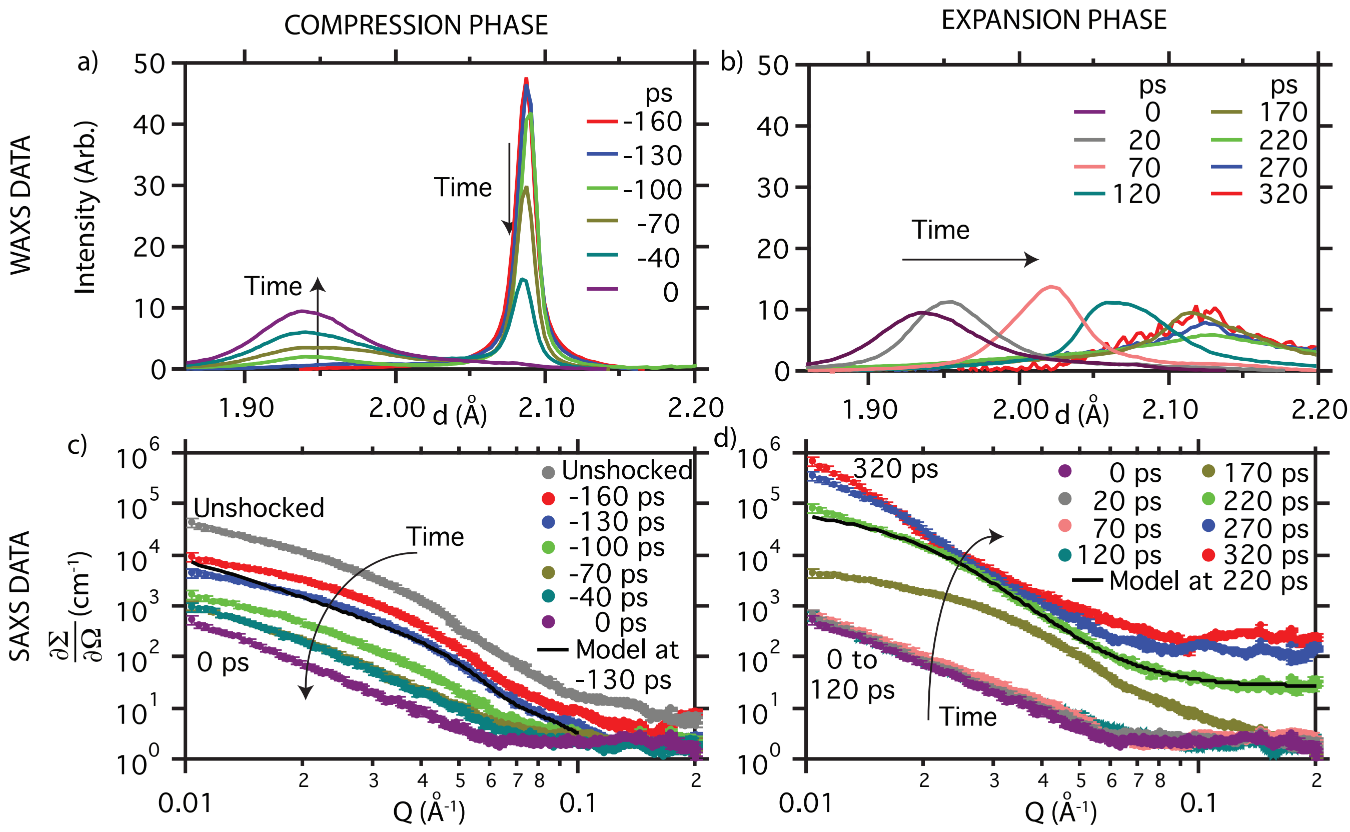

Fig. S. 2: **Radially integrated scattering data illustrating the evolution of WAXS and SAXS profiles with time**. **a, b)** The evolution of WAXS and **c, d)** SAXS profiles, corresponding to a, c) strain induced sample compression and b, d) subsequent sample expansion. A SAXS model fit is shown at -130 ps in (b) and 220 ps in (d). t_0 at 0 ps refers to the time in the shock event at which the sample was fully compressed.
